# Protocol for predicting suppressors of cell-death pathways based on transcriptomic and vulnerability data

**DOI:** 10.1016/j.xpro.2025.103855

**Published:** 2025-05-29

**Authors:** Yaron Vinik, Avi Maimon, Sima Lev

**Affiliations:** 1 Molecular Cell Biology Department, Weizmann Institute of Science, Rehovot 76100, Israel

**Keywords:** Bioinformatics, Cancer, RNAseq

## Abstract

Here, we present a computational protocol for predicting repressors of cancer cell death by combining vulnerability and transcriptomic responses to cell death inducers. We describe steps for calculating a set of predictors for each gene and aggregating them into a single metric, ranking the genes according to their predictive power. We then detail procedures for selecting candidate genes for experimental validation based on this ranking and other considerations. This protocol enables the identification of several experimentally validated cell death repressors.

For complete details on the use and execution of this protocol, please refer to Vinik et al.^1^

## Highlights

• Computational approach for discovering cell death suppressors
• Guidelines for measuring and combining gene-level predictors
• Guidelines for selecting potential targets based on the measured predictors

## Abstract (editor highlights)

Publisher’s note: Undertaking any experimental protocol requires adherence to local institutional guidelines for laboratory safety and ethics.

## Graphical abstract

**Figure:**
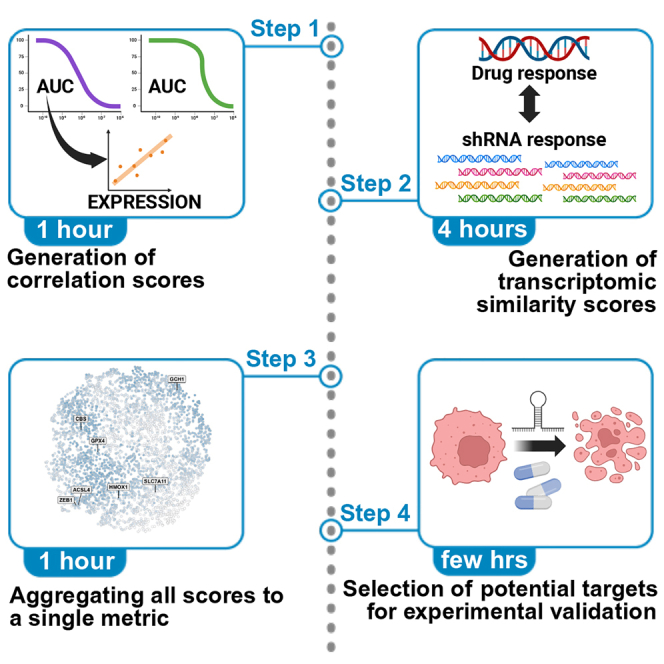

## Before you begin

In this protocol, we merge two drug discovery approaches to reliably predict candidate genes that suppress cancer cell death pathways. In the first approach, we use a set of cell death inducers to define vulnerabilities across multiple cancer cell lines, and then correlate the vulnerabilities to public datasets of gene expression and gene essentiality in cancer cell lines. High correlations highlight genes whose targeting may increase vulnerability to the death pathway. In the second approach, we profile the transcriptomic response of the death inducers by RNA sequencing in several breast cancer cell lines. We then compare these transcriptomic responses to the transcriptomic response signatures induced by gene silencing using public datasets of thousands of transcriptomic signatures (Connectivity Map; CMAP, Broad Inst.). We quantify the transcriptomic similarity and rank the genes according to their similarity scores. Subsequently, we merge the two approaches and generate a single metric that measures the potential of each gene to be a death repressor and consequently a possible target for cancer therapy. Two methods have been used for merging: (i) aggregating the scores of the two approaches into a single metric, and (ii) using a dimension reduction method to project all the scores on a 2D plane and measure the distances between the genes and a central key gene of the pathway. Throughout this protocol, we employed several computational validation approaches, and subsequently experimentally validate the selected genes.

This protocol could be powerful for any drug that induce cell death. The inputs are (i) the vulnerability of the cell lines to the drug which can be measured by dose response curves, and (ii) the transcriptomic response profile of the drug based on RNA-seq data. The described computational pipeline looks for genes whose targeting would mimic the vulnerability and the transcriptomic responses of the death inducing drugs in the cancer cell lines. This can offer alternative druggable targets for drugs which are very effective in vitro but somehow not effective and/or are highly toxic in vivo. It may also improve the efficacy of a given drug and even overcome resistance by targeting a bypassed pathway that eventually leads to the same death response.

### Preparation one: Generate dose response curves

**Timing: 5 days**

1. In 96-well plates, seed the cells one day before treatment. Count the number of cells and maintain constant conditions in all experiments.

**CRITICAL:** The confluency of the cells greatly affects their response to the drugs and should not exceed 50% on the day of treatment. The number of cells to seed per well should be determined for each cell line, depending on their growth rate and relative size.

2. 24 h later, replace the medium with medium containing increasing doses of the drug(s) to generate a dose response curve.

***Note:*** The concentrations range should be experimentally determined for each drug and might be varied between different drugs suppliers. This range should be suitable for most of the cell lines with close to 100% viability at the lowest concentration tested and below 25% viability in the highest concentration tested. A preliminary experiment can be useful to determine this range. The experiment should be done in triplicates.

**CRITICAL:** If the drug is soluble in DMSO (or any other solvent), it is important to maintain identical volumes of DMSO in all drug concentrations. Wells with untreated cells (which include the same volume of DMSO but without drug) should also be included. DMSO concentrations should not exceed 0.1% (v/v) of the total media volume to ensure minimal interference with cell growth and viability.

3. Incubate the cells in their optimal growth conditions (usually in a 37°C humidified incubator of 5% CO_2_).
4. 72 h post-treatment, perform MTT assay (or a similar method^2^) to determine cell viability.

For each concentration, calculate the average cell viability of the repeats, and compare this average to the viability of the control non-treated cells. Results should be given in % viability vs. the control cells.

### Preparation two: Generate transcriptomic response data

**Timing: 2 weeks**

5. Use the dose response curves performed in Preparation one to determine the IC_50_ values for each drug in each cell line. To that end, use the code provided in step 1b below.
6. Seed the cell lines in 6 well plates (adjust cell number to accommodate 50% confluency at the day of treatment).

**CRITICAL:** The confluency of the cells is critical and greatly affects the results.

7. 24 h later, replace the medium with medium containing the drugs, using the IC_50_ concentration as determined in step 5. Further, prepare vehicle control by adding the same volume of the solvent (such as DMSO) to untreated cells.

**CRITICAL:** At least 3 repeats are needed.

8. 4–24 h later, extract RNA using standard methods (such as Trizol) and then perform RNA sequencing.

***Note:*** The exact time point should be determined in a preliminary time points experiment to ensure a broad capturing of the transcriptomic response. A qRT-PCR analysis of typical biomarkers of the induced death pathway can help to determine the optimal time point. Example of time dependence transcriptomic response can be seen in Figure S3C in Vinik et al.^3^

***Note:*** The output of this step should be a count matrix, consisting of genes in rows and treatments in columns.

### Preparation three: Install R and associated packages

**Timing: 1 h**

9. Download and install R 4.4.0 (https://cran.r-project.org/).
10. Download and install RStudio (https://www.rstudio.com/).
11. Install the required packages from CRAN (https://cran.r-project.org/) or Bioconductor (https://bioconductor.org/) in RStudio using this code:

#install and load libraries

install.packages(tidyverse, drc, cmapR, umap, rentrez, ggrepel)

if (!require("BiocManager", quietly = TRUE))

 
install.packages("BiocManager")

BiocManager::install("PharmacoGx")

BiocManager::install("limma")

BiocManager::install("cogena")

BiocManager::install("fgsea")

library(tidyverse)

library(drc)

library(PharmacoGx)

library(cmapR)

library(umap)

library(rentrez)

library(limma)

library(cogena)

library(fgsea)

library(ggrepel)

## Key resources table

**Table:** 

REAGENT or RESOURCE	SOURCE	IDENTIFIER
**Deposited data**		

Code generated in this study	This paper	Zenodo link: https://doi.org/10.5281/zenodo.13370886 GitHub link: https://github.com/SimaLevLab/Pipeline-for-discovery-of-ferroptosis-targets

**Software and algorithms**		

R version 4.4.0	CRAN R project	https://www.r-project.org/
RStudio version 2024.04.2	Posit	https://posit.co/download/rstudio-desktop/
tidyverse package, v.2.0.0	N/A	https://www.tidyverse.org/
drc package, v.3.0	N/A	https://cran.r-project.org/web/packages/drc/index.html
pharmacoGx package, v.3.8.0	N/A	https://www.bioconductor.org/packages/release/bioc/html/PharmacoGx.html
cmapR package, v.1.16.0	N/A	https://www.bioconductor.org/packages/release/bioc/html/cmapR.html
umap package, v.0.2.10.0	N/A	https://cran.r-project.org/web/packages/umap/index.html
rentrez package, v.1.2.3	N/A	https://cran.r-project.org/web/packages/rentrez/index.html
limma package, v.3.60.2	N/A	https://bioconductor.org/packages/release/bioc/html/limma.html
cogena package, v.1.38.0	N/A	https://www.bioconductor.org/packages/release/bioc/html/cogena.html
fgsea package, 1.30.0	N/A	https://bioconductor.org/packages/release/bioc/html/fgsea.html
ggrepel package, 0.9.5	N/A	https://cran.r-project.org/web/packages/ggrepel/index.html

**Other**		

Hardware	N/A	We recommend a machine with at least 32 GB, intel i5-5900 CPU, or equivalent.

## Step-by-step method details

The following steps include code snippets relevant for each step. To view the entire code, with working example (in which we search for ferroptosis related drug targets), please see our code in GitHub (https://github.com/SimaLevLab/Pipeline-for-discovery-of-ferroptosis-targets) or Zenodo (https://doi.org/10.5281/zenodo.13370886).

### Generation of the correlation scores

**Timing: 1 h**

In this step, the correlation predictor values will be determined based on the calculated area under the curves (AUCs) from the dose response curves described in preparation step 1.

1. Calculate the AUCs of the dose response curves.
  a. Arrange the dose response data (see preparation step 1) as shown in Table 1.

**Table 1:** An example of how to organize the dose response data

Cell_line	Concentration	logConc	Repeat_1	Repeat_2	Repeat_3
MDAMB468	5.00E-08	−7.30103	100	100	100
MDAMB468	3E-07	−6.52288	70.57607	66.23268	53.81605
MDAMB468	6E-07	−6.22185	61.12453	42.88114	51.27202
MDAMB468	1.2E-06	−5.92082	50.81063	41.32718	45.79256
MDAMB468	2.4E-06	−5.61979	47.70611	35.1533	48.53229
MDAMB468	4.8E-06	−5.31876	39.66885	31.12138	43.05284
MDAMB468	9.6E-06	−5.01773	29.32046	21.79756	31.89824
MDAMB468	1.92E-05	−4.7167	20.38634	15.58169	14.48141

The “Repeat_X” columns depict the cell line viability (% of controls). “logConc” is the concentration in a logarithmic base 10 scale.
  b. Calculate the AUC using the following code in R:
    #In the code below, "DRC_data" is a dataframe organized as shown in table 1 (step 1a).
    DRC_data <- read_csv("../Data/Star Protocol - DRC data.csv")
    DRC_measures <- DRC_data %>%
     dplyr::select(-Concentration) %>%
     gather(-Cell_line, -logConc, key = "rep", value = "viability") %>%
     na.omit()
    drmModel <- drm(viability ∼ logConc, data = DRC_measures, curveid = rep,
       fct = L.4(names = c("slope", "lower limit", "upper limit", "EC50"),
         fixed = c(NA, 0, 100, NA)))
    result <- drmModel[["parmMat"]] %>%
     t() %>%
     as_tibble(rownames = "rep") %>%
     dplyr::rename("slope" = "V1", "IC50" = "V2")
    aucs <- DRC_measures %>%
     group_by(rep) %>%
     summarise(auc = 100 - computeAUC(logConc, viability, conc_as_log = TRUE, viability_as_pct = TRUE, area.type = "Actual"))
    parameters <- left_join(result, aucs, by = "rep")
  c. Gather the AUCs of all cell lines in one data frame, as shown in Table 2.

**Table 2:** An example of how to organize the AUC data

Cell_line	Inducer 1	Inducer 2	Inducer 3
BT20	54.961	55.145	63.318
BT474	95.609	96.487	79.182
BT549	51.413	69.378	65.539
HCC1143	74.919	79.182	96.487
HCC1937	60.041	80.197	75.877
HCC70	63.664	71.355	78.426
T47D	74.412	79.839	95.065
2. From the DepMap portal (https://depmap.org/portal/data_page/?tab=allData), download the most updated version of the following files:
  a. OmicsExpressionProteinCodingGenesTPMLogp1.csv: RNA sequencing of cancer cell lines. Gene expression is given as TPM (transcript per million) values.
  b. CRISPRGeneEffect.csv: CRISPR screen for gene dependency. Gene effect is given in a score ranging from 0 (median of non-essential genes) to −1 (median of essential genes).
  c. Model.csv: List of cell lines and models used in the DepMap project.
3. In R, combine the AUC data with the TPM or Gene effect data by cell lines, and calculate the Pearson’s correlation between each gene in the TPM or Gene effect data and the AUCs data, using the code below.

***Note:*** Make sure that the cell lines names in the AUC data file match exactly with the cell lines names in the DepMap files, in order to merge the files correctly. Use the Model.csv file to translate the cell lines identifiers (ACH-XXXXXX) to the actual cell lines names.

#AUCs – data frame containing the AUCs in the format shown in table 2 (step 1c).

#TPM - the TPM data for all genes in the relevant cell lines, downloaded from DepMap.

Expression_corrMatrix <- AUCs %>%

 left_join(TPMs, by = c("Cell_line")) %>%

 dplyr::select(-`Cell_line`) %>%

 cor(method = "pearson", use = "complete.obs") %>%

 as_tibble(rownames = "gene") %>%

 dplyr::select(gene, `Inducer 1`, `Inducer 2`, `Inducer 3`) %>%

 filter(!(gene %in% c("Inducer 1", "Inducer 2", "Inducer 3"))) %>%

 na.omit()

4. For each gene, combine the two predictors generated per inducer (the correlation values between the inducer AUC and the gene TPM or gene effect) into a single dataframe.

**Figure 1:**
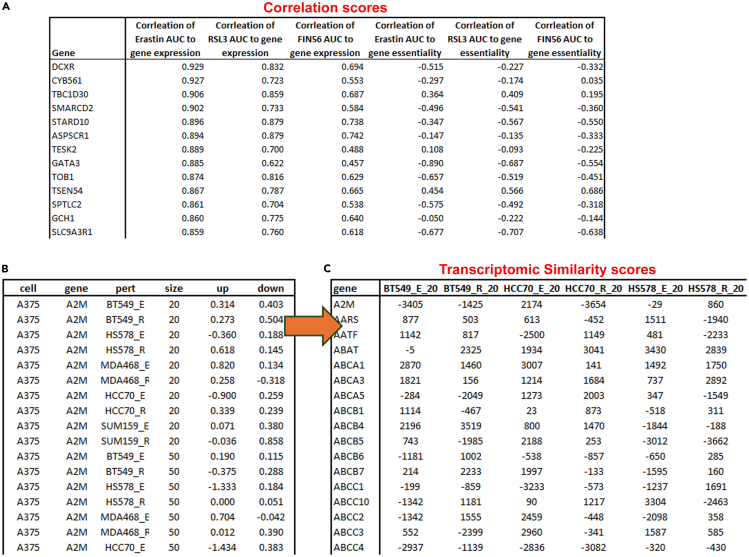
Correlation and transcriptomic similarity scores The pipeline generates several scores for each gene, based on the input data. (A) shows the correlation scores (correlation between gene expression or essentiality to drug AUCs), as calculated in step 4. (B) shows the transcriptomic similarity scores. Here, the transcriptomic signatures induced by different perturbations (shown in the “pert” column) are compared to signatures in the Connectivity Map, in which shRNAs were used to knock-down ∼4000 genes (in the “gene” column) in different cell lines (shown in the “cell” column). For each gene + cell line combination, there are two similarity scores: for the most upregulated and the most downregulated genes in each signature (shown in the “up” and “down” column). This table is the result of step 8. Step 9 is then used to aggregate all the connectivity map scores for each gene, creating one score for each gene and each perturbation (shown in C).

***Note:*** An example of the outcome is shown in Figure 1A.

### Generation of the transcriptome similarity scores

**Timing: 4 h**

In this step, the transcriptomic similarity predictor values will be determined for each gene. To this end, the transcriptomic signatures in response to the death inducing drugs are compared to the transcriptomic signatures of gene knock-down response.

5. Using the count matrix created in preparation step 2, perform gene expression normalization in R and determine the differential gene expression between each drug and its vehicle control in each cell line.

***Note:*** This can be done by tools such as DESQ2 or edgeR. For an example of analysis using DESQ2, see Sanchis et al.^4^ For an example of using edgeR, see our code here: https://doi.org/10.5281/zenodo.13370886.

***Note:*** The result of this analysis should be a vector of the fold changes in the expression of all genes between the drug treated and the control cells. Such a vector should be acquired for each combination of drug and cell lines. Henceforth they will be termed “drug response signatures”.

6. Download the connectivity map (CMAP) data from https://www.ncbi.nlm.nih.gov/geo/query/acc.cgi?acc=GSE92742. These are the necessary databases from this GEO record:
  a. GSE92742_Broad_LINCS_Level5_COMPZ.MODZ_n473647x12328.gctx (signatures file – note this file is ∼20 GB).
  b. GSE92742_Broad_LINCS_sig_info.txt (signatures meta-data).
  c. GSE92742_Broad_LINCS_gene_info.csv (genes meta-data).

***Note:*** The CMAP data downloaded in this step include transcriptomic signatures in several cancer cell lines, in response to various compounds and gene perturbations (knock-down, knock-out and over expression).

7. Run the following code, which filters only the shRNA signatures, and extracts from each signature the top 20 up- or downregulated genes in response to each shRNA in every cell line. These signatures will be saved in a GMT format.

***Note:*** The approximate runtime of this code is ∼2.5 h.

# Load CMAP data

ds_path <- "Data/GSE92742_Broad_LINCS_Level5_COMPZ.MODZ_n473647x12328.gctx" #path to the signature file

sig_meta <- read.delim("Data/GSE92742_Broad_LINCS_sig_info.txt") #path to signatures meta-data

gene_data <- read.csv("Data/GSE92742_Broad_LINCS_gene_info.csv") #path to genes meta-data

# Filter signature metadata to select IDs related to a specific perturbation type (the shRNA screen)

relevant_IDs <- sig_meta %>%

 filter(pert_type == "trt_sh.cgs") %>%

 pull(sig_id)

# Load column metadata from the GCTX file and filter based on relevant IDs

col_meta <- read_gctx_meta(ds_path, dim="col")

idx <- which(col_meta$id %in% relevant_IDs)

# Parse the GCTX file to extract only the relevant columns (filtered by idx)

my_ds <- parse_gctx(ds_path, cid = idx)

x1 <- my_ds@mat

x1 <- as_tibble(x1, rownames = "ID")

# Initialize lists to store gene sets for top and bottom 20 genes

geneset_size <- 20

TS_geneset_list_up <- list()

TS_geneset_list_down <- list()

# Loop through each signature in the data matrix, retrieving the 20 genes with the highest and lowest fold change

for(n in 1:dim(x1)[2]) {

 currCol <- colnames(x1)[n+1] # Get the name of the current column (signature ID)

 X1DF <- x1 %>%

  select(ID, any_of(currCol)) %>%

  arrange(desc(across(any_of(currCol)))) %>%

  mutate(ID = as.numeric(ID)) %>%

  left_join(gene_data, by = "ID") %>%

  dplyr::select(name)

 TS_geneset_list_up[n] <- slice_head(X1DF, n = geneset_size)

 TS_geneset_list_down[n] <- slice_tail(X1DF, n = geneset_size)

 names(TS_geneset_list_up)[n] <- currCol

 names(TS_geneset_list_down)[n] <- currCol

 # Print progress

 print(paste0("creating: ", n, " / ", dim(x1)[2], " (", round(n/dim(x1)[2]∗100,3), "%)"))

}

# Export each gene set list to a GMT file for different top/bottom N gene sets

gmtlist2file(TS_geneset_list_up, "CMAP TS complete gene set up (20 genes).gmt")

gmtlist2file(TS_geneset_list_down, "CMAP TS complete gene set down (20 genes).gmt")

8. Determine the enrichment of each gene set extracted from CMAP (step 7) in each of the drug response signatures (step 5).

***Note:*** This step can be performed by several enrichment methods. We have implemented the Camera method (see for example our code in https://doi.org/10.5281/zenodo.13370886). Below is a code implementing the Camera method. Prior to running this code, the user should apply the voom() function from Limma to create a voom object, which should be stored in the “V” variable. Further, the “design” and “cont.matrix” should hold the design matrix and contrasts matrix, respectively. For further information about the “voom” object, see Law et al.,^5^ and our example code in https://doi.org/10.5281/zenodo.13370886.

***Note:*** The outcome of this step is shown in Figure 1B.

CameraFunction <- function(contr.matrix, V, idx, design) {

 cam.list <- list()

 for(n in 1:dim(contr.matrix)[2]) {

 name <- names(contr.matrix[,n])[contr.matrix[,n] == 1]

 cam.list[[n]] <- camera(V, idx, design, contrast=contr.matrix[,n]) %>%

 as_tibble(rownames = "pathway") %>%

 mutate(result = ifelse(Direction == "Up", PValue, -PValue)) %>%

 dplyr::select(pathway, result)

 names(cam.list[[n]])[names(cam.list[[n]]) == "result"] <- name

 print(n)

 }

 cam.object <- reduce(full_join, cam.list)

 cam.object.log <- cam.object %>%

 mutate(across(where(is.numeric), ∼ifelse(.x>0, -log10(.x), log10(abs(.x)))))

 return(cam.object.log)

}

GMT_files <- c("CMAP TS complete gene set up (20 genes).gmt",

 "CMAP TS complete gene set down (20 genes).gmt")

Camera_result <- tibble("pathway" = names(gmt2list(paste0("Data/", GMT_files[1]))))

for (n in 1:2) {

 title <- str_extract(GMT_files[n], "gene set [:alpha:]∗ \\([:digit:]∗ genes\\)")

 title_short <- paste(str_extract(title, "up|down"), str_extract(title, "[:digit:]∗(?= genes)"), sep = "_")

 CMAP_geneset <- gmt2list(paste0("Data/", GMT_files[n]))

 idx <- ids2indices(CMAP_geneset, identifiers = V$genes$genes)

 x <- CameraFunction(contr.matrix, V, design, idx) %>%

 rename_with(∼paste0(.x, "_", title_short), .cols = -pathway)

 Camera_result <- Camera_result %>% left_join(x1)

}

CMAP_analysis <- Camera_result %>%

 mutate(ID = str_replace(pathway, "ˆCGS[:digit:]∗_", ""),

   ID = str_replace(ID, "_[:alnum:]∗:", "_"),

   ID = str_replace(ID, ":[:graph:]∗$", "")) %>%

 filter(str_detect(pathway, "96H")) %>%

 dplyr::select(ID, everything(), -pathway)

#create a long version of the "CMAP analysis" object for further work

CMAP_analysis_long <- CMAP_analysis %>%

 mutate(cell = sapply(str_split(ID, "_"), function(x) x[1])) %>%

 mutate(gene = sapply(str_split(ID, "_"), function(x) x[2])) %>%

 select(-ID) %>%

 pivot_longer(-c(cell, gene), names_to = c("pert", ".value", "size"), names_pattern = "(.∗_.)_(.∗)_(.∗)")

9. Calculate the transcriptomic similarity predictors for each gene by performing the following:
  ***Note:*** The shRNA screen in CMAP was performed for several cell lines (4–9). Each shRNA in each cell line produces two enrichment scores, as calculated in step 8 – one for the upregulated gene set and one for the downregulated gene sets.
  a. For each shRNA, aggregate the 4-9 scores achieved for the different CMAP cell lines, by taking their maximum value. This step results in two scores for each shRNA (one for the upregulated gene sets and one for the downregulated gene sets), for every drug response signature (see step 9a in the code below).
  b. In each drug response signature rank the scores obtained for each shRNAs (separately for the up- and down- regulated gene sets), starting with 1 for the shRNA with the lowest score (see step 9b in the code below).
  c. For each gene (shRNA), subtract the rank of the downregulated score from the rank of the upregulated score. This will generate one score, representing the transcriptomic similarity between the gene KD response signatures and the drug response signatures (see step 9c in the code below).
    ***Note:*** The outcome of this step is shown in Figure 1C.
    #aggregate CMAP scores. In the code below, "CMAP_analysis" is the dataframe created
    #in the code snippet attached to step 8.
    #Step 9a: Each gene has 4-9 scores (one for each of the cell lines used in CMAP).
    # Get for each gene the maximum value.
    CMAP_analysis_max <- CMAP_analysis %>%
     mutate(cell = sapply(str_split(ID, "_"), function(x) x[1])) %>%
     mutate(gene = sapply(str_split(ID, "_"), function(x) x[2])) %>%
     dplyr::select(ID, cell, gene, everything()) %>%
     group_by(gene) %>%
     summarise(across(contains("_"), max))
    #Step 9b: Rank the genes based on their aggregated scores
    #(for upregulated and downregulated signatures separately)
    CMAP_analysis_max_ranked <- CMAP_analysis_max %>%
     mutate(across(contains("_"), ∼min_rank(.x)))
    #Step 9c: Combine the upregulated and downregulated signatures.
    # The final metric diff is UP - DOWN, so -4000 is the best, +4000 is the worse
    CMAP_analysis_max_combined <- CMAP_analysis_max_ranked %>%
     gather(-gene, key = "pert", value = "rank") %>%
     mutate(up_down = str_extract(pert, "up|down"), .after = pert) %>%
     mutate(pert = str_remove(pert, "_up|_down")) %>%
     spread(key = "up_down", value = "rank") %>%
     mutate(total = up - down) %>%
     dplyr::select(-up, -down) %>%
     spread(key = "pert", value = "total")

10. Perform validation of the transcriptomic similarity scores.
  ***Note:*** This optional validation step can be carried out providing that existing knowledge concerning the drug-inducing death pathways is known. This validation relies on the following:
  a. Known target genes: If the drug in question has a known target gene, the similarity score for that gene should be high. For example, we previously showed that GPX4 has high transcriptomic similarity score in RSL3 treated cells, as GPX4 is the target of RSL3 (see Figure 4C in Vinik et al.^1^). However, this is not always the case, especially if a gene is increased in response to the drug that inhibits its protein product as a protective mechanism. For each such key gene, it is recommended to examine the calculated score for all drug response signatures and apply another filtration step if the scores are too variant (see problem 1 in the troubleshooting section for details).
  b. Text-mining: In this approach, the transcriptomic similarity scores for each gene are aggregated (for example, by average), and the top 100 genes with the highest average are queried in PubMed with terms specific for the pathway in question. The code in step 16b below describes how to perform automated PubMed searches within R. The number of cited genes with the pathway specific term among the top 100 genes with the highest transcriptomic similarity scores can be compared to randomly selected 100 genes analyzed in the same way.

### Ranking potential targets by “best predictors” method

**Timing: 1 h**

Following the previous two sections, each gene should have a set of correlation predictors and transcriptomic similarity predictors. To rank the genes by their potential to induce the death pathway upon targeting, two methods can be applied, the method described below (Steps 11–13) or in the next section (Steps 14–15). In this section, the genes are ranked by their predictor values. This procedure doesn’t require pre-existing knowledge concerning the pathway in question.

11. Combine the correlation predictors (Step 4) and transcriptomic similarity predictors (Step 9) into one data frame and present all the predictors for each gene as shown in Table 3.

**Table 3:** An example of how to organize the predictors data

Gene	Inducer 1 AUC cor to expression	Inducer 2 AUC cor to expression	Inducer 1 AUC cor to depscore	Inducer 2 AUC cor to depscore	Transcriptomic similarity to HCC70 treated with inducer 1	Transcriptomic similarity to HCC70 treated with inducer 2	Transcriptomic similarity to BT549 treated with inducer 1	Transcriptomic similarity to BT549 treated with inducer 2
CYP1B1	−0.279	−0.234	−0.049	−0.102	−3783	1074	−35	−1640
ZNF180	0.014	−0.178	−0.444	−0.535	−3700	959	114	−1210
CLK1	−0.222	−0.355	−0.307	−0.318	−3652	−1115	−393	−2012
SLC25A46	−0.565	−0.615	−0.457	−0.398	−3621	−2850	−2886	−876
RPTOR	−0.074	0.063	0.012	0.028	−3441	424	629	3020
PDLIM1	−0.418	−0.445	−0.330	−0.275	−3417	−1830	−198	−118
BAZ1B	−0.316	−0.108	0.097	0.010	−3387	1970	1896	743
12. Aggregate all the predictor scores into one score for each gene. This is one method to do it (implemented in the code snippet shown below):
  a. Calculate the average of all correlations between the inducers AUCs and gene expression. Rank these averages.
  b. Calculate the average of all correlations between the inducers AUCs and gene effect (essentiality). Rank these averages.
  c. For each gene, take the maximum value of its ranks calculated in steps a and b.
  d. Calculate the average of all transcriptomic similarity scores for each gene. Rank these averages.
  e. For each gene, calculate the average of the rank from step c and the rank from step d.
  f. Rank the average of the scores calculated in e.

#Corrlation - hold the correlation predictors for each gene

#CMAP - contains the transcriptomic similarity scores per gene

GeneList <- full_join(Correlations, CMAP, by = "gene")

best_predictors_rank <- GeneList %>%

 mutate(mean_r_expression = rowMeans(across(contains("AUC cor to expression"))),

   mean_r_depscore = rowMeans(across(contains("AUC cor to depscore"))),

   biggest_r = pmax(mean_r_expression, mean_r_depscore, na.rm = TRUE),

   mean_CMAP_scores = rowMeans(across(contains("transcriptomic similarity")))) %>%

dplyr::select(gene, mean_r_expression, mean_r_depscore, biggest_r, mean_CMAP_scores) %>%

filter(!is.na(mean_CMAP_scores)) %>%

mutate(rank_corr_expression = min_rank(mean_r_expression),

   rank_corr_depscore= min_rank(mean_r_depscore),

   rank_corr = pmax(rank_corr_expression, rank_corr_depscore, na.rm = TRUE),

   rank_CMAP_score = min_rank(mean_CMAP_scores),

   mean_rank = (rank_corr + rank_CMAP_score) / 2,

  rank_total = min_rank(mean_rank))

13. Observe the top-ranking genes (genes with the highest “rank_total”, if using the code above). These genes should be populated with potential target genes.

**Figure 2:**
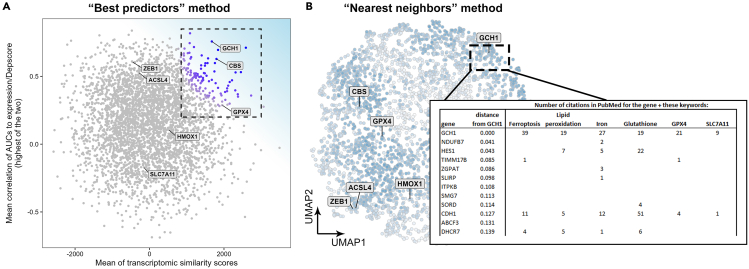
Methods for ranking genes based on their potential to be suppressors of death pathways Two methods were developed. In the “Best predictors” method (described in steps 11–13), the correlation and transcriptomic similarity scores are aggregated into one score. The scatterplot (A) shows two intermediate scores, summarizing all the correlation scores (measured in step 12c, shown in the y-axis) and the transcriptomic similarity scores (measured in step 12d, shown in the x-axis). The top left corner should include the genes with the best predictor values (A). In the “Nearest neighbors” method (described in steps 14-15), UMAP is used to generate a 2D map of all genes, in which proximity between genes represents proximity in the predictor values (B). The genes are then ranked by their distance from important nodes (in this example, GCH1, which is a major anti-ferroptosis gene). Automated PubMed searches are then performed using the gene names and pathway specific terms, in order to identify genes which were not previously published with those terms. The panels in this figure are based on figures published in our previous paper (Vinik et al.^1^).

***Note:*** An example for the outcome of this section is shown in Figure 2A (taken from Vinik et al.^1^). This plot highlights the genes with the highest ranks as calculated in step 12c (in the y-axis) and 12d (in the x-axis), revealing several high-scoring genes related to ferroptosis in the top-right corner.

### Ranking potential targets by nearest neighbors method

**Timing: 1 h**

This step involves projecting all predictors on a 2D plane using dimension reduction methods, identifying a key gene in the pathway, and ranking all other genes based on their distance to that gene on the plane.

14. Combine all predictors to a single data frame, as done in step 11, and perform a dimension reduction by the UMAP method using the code below.

***Note:*** The outcome of this step is shown in Figure 2B.

#clustering by UMAP

#Corrlation - hold the correlation predictors for each gene

#CMAP - contains the transcriptomic similarity scores per gene

set.seed(42)

GeneList <- full_join(Correlations, CMAP, by = "gene")

thisDF.prep <- GeneList %>%

 arrange(gene) %>%

 na.omit() %>%

 mutate(across(where(is.numeric), scale))

thisDF.data <- thisDF.prep %>% select(where(is.numeric))

thisDF.labels <- thisDF.prep %>% select(gene)

thisDF.map <- umap(thisDF.data, preserve.seed = TRUE, random_state = 5023, min_dist = 0.05)

result <- cbind(thisDF.labels, thisDF.map$layout)

# in the following line, replace the genes with the key genes in the desired pathways.

# currently this line shows key genes in ferroptosis.

selected_labels <- c("GPX4", "SLC7A11", "HMOX1", "ACSL4", "CBS", "ZEB1", "GCH1")

ggplot(result, aes(x = `1`, y = `2`)) +

 geom_point(size = 2, shape = 21, alpha = 0.8, color = "gray") +

 geom_label_repel(data = result %>% filter(gene %in% selected_labels), aes(label = gene),

 size = 4, color = "black", fill = "gray90", fontface = "bold") +

 labs(x = "", y = "") +

 theme_bw() +

 theme(legend.position = "none",

 panel.grid = element_blank())

15. Identify a key gene(s) in the inquired pathway and measure the Euclidean distances of all genes from that node. Then, rank all genes based on this distance. The following function performs this analysis.

***Note:*** An example of the output of this code is shown in Figure 2B (inset table).

#"result" – the UMAP result from the previous code snippet.

nodes_nearest_neighbors <- function(zoom_on_gene) {

 coord.x <- result[result$gene == zoom_on_gene, ]$`1`

 coord.y <- result[result$gene == zoom_on_gene, ]$`2`

 neighborhood <- 0.4

 node_neighbors <- result %>%

 mutate(distance = sqrt((`1` - coord.x)ˆ2 + (`2` - coord.y)ˆ2)) %>%

 select(gene, distance) %>%

 arrange(distance) %>%

 slice_min(distance, n = 75) %>%

 left_join(GeneList, by = "gene")

}

### Select genes from the potential target list for further analysis

**Timing: a few hours**

In step 13 or step 15, a ranking of the genes is made based on their predictor values. In this step, the selection of the genes for further analysis will be carried out based on several factors.

16. Observe the ranked list of genes achieved in steps 13 or step 15. Select potential targets for experimental validation using the following criteria:
  a. The ranking in the list should be the most important criteria. Consider the top 75 ranked genes.
  b. Target novelty: an automated PubMed search can be used to filter out genes that already have citations related to the pathway. The following function receives a list of genes (for example, the top 75 most ranked genes from steps 13 and 15), and a list of terms related to the pathway in question.
    ***Note:*** The outcome of this step is shown in the table in Figure 2B (inset table).
    #pubmed search
    #Pubmed_terms should include terms related to the pathway in question
    Pubmed_terms <- c("Ferroptosis", "Lipid peroxidation", "Iron", "Glutathione", "GPX4", "SLC7A11")
    #In the next line, replace "PUBMED_KEY" with your personal pubmed access key,
    #Which can be received in the NCBI site
    set_entrez_key("PUBMED_KEY")
    get_pubmed_info <- function(genes, Pubmed_terms) {
     pubmed_search <- list()
     for(i in seq_along(genes)) {
     print(genes[i])
     for(j in seq_along(Pubmed_terms)) {
     print(str_c("---", Pubmed_terms[j]))
     search_term <- paste0(genes[i], " AND ", Pubmed_terms[j])
     pubmed_result <- entrez_search(db = "pubmed", term = search_term)
     pubmed_search[[i∗10 + j]] <- tibble("gene" = genes[i], "term" = Pubmed_terms[j], "count" = pubmed_result$count)
     }
     Sys.sleep(0.1)
     }
     pubmed_search_tib <- purrr::reduce(pubmed_search, bind_rows)
    }
    #In the line below, "genelist" is a vector with the gene names that will be inquired for in
    #PubMed. This could be a result of steps 13 or 15 in this protocol.
    pubmed_data <- get_pubmed_info(as.vector(genelist), Pubmed_terms)
    save(pubmed_data, file = "Pubmed search for ranked list.RData")
    ***Optional:*** To focus only on druggable targets, the list of genes can be filtered for druggability and toxicity using the information from the DepMap site (https://depmap.org/portal/). Druggability information is given for each gene in the “overview” page. Toxicity can be considered for genes which are “common essential”, also shown in the “overview” page.
17. Validate experimentally the selected genes, using gene targeting (for example, by shRNA, siRNA, sgRNA) and examine the hallmarks of the relevant pathway.

***Note:*** This step depends on the relevant pathway. As an example, see Figure 5 in our previous publication (Vinik et al.^1^).

## Expected outcomes

The outcome of either step 13 or 15 are lists of genes, ranked by their potential to be efficient targets that induce the desired death phenotype. Step 16 is used to filter these lists to accommodate non-computational requirements, such as druggability and toxicity. The overall outcome is a set of candidate genes, which can then be validated experimentally.

## Quantification and statistical analysis

The ranked list of genes achieved in steps 13 and 15 are supposed to be populated by genes whose targeting should induce the desired phenotype. Statistical examination of this statement is challenging, but can be addressed using the following approach: we downloaded the pan-cancer public data of gene expression (CCLE, taken from the DepMap site) and drug sensitivity data (such as CTRP, also from the DepMap site), and calculated the correlations between the expression of each gene and the sensitivity of the cells to the drugs in question. Then, the correlations of the top-ranking genes are examined against the backdrop of all genes using GSEA. In the following code, “gene_list” is an example of 75 genes which were ranked high as potential ferroptosis targets. The enrichment of the correlations between their expression and ferroptosis drug sensitivity is measured and is then compared to the correlations of all other genes. See problem 2 in the troubleshooting section if such enrichment cannot be found.

#GSEA validation

gene_list <- list("Top_ranking_genes" = c("GCH1","NDUFB7","HES1","TIMM17B","ZGPAT","SLIRP","ITPKB","SMG7","SORD","CDH1","ABCF3","DHCR7",

"CHMP2A","MYBL2","CEBPA","ACAA1","BZW2","PIK3C2B","ZBTB46","BATF","CANT1","ZNF263","HDAC11",

"AKT1","DNAJC17","ERBB2","SDF2L1","ARRB1","UBE4A","NDUFV1","NLK","GPR26","GREB1","ALDH6A1","FASN",

"DECR1","F7","GCAT","TLE2","ALDH7A1","TNFRSF18","FRAT1","PPP2R5A","DCXR","ELF3","SPTLC2","RHOB",

"BLZF1","TBX2","RIPK2","MAP3K9","NDUFC2","ZNF629","DEPTOR","PIPOX","PIK3R3","LRSAM1","KSR2","NF1",

"NDUFA6","BLNK","CSRP1","MAPK3","ATP6V0A1","PON3","ALDOC","AXIN1","PRKD2","ZNF217","MTHFD2","CNOT3",

"DNPEP","NAT1","B4GALT3","ANO1"))

# Load CCLE data (cell line expression data) - download from Depmap

CCLE_pan <- read_csv("../Data/CCLE pan 20Q4.csv")

# Load CTRP data (drug response data) - download from DepMap

CTRP_pan <- read_csv("../Data/CTRP pan AUCs.csv") %>%

 separate(CCLE_Name, into = c("ID", "Lineage", sep = "_"))

# Define a vector of ferroptosis-inducing drugs that can be found in CTRP

Drugs <- c("erastin", "ML210", "ML162")

#Merge CCLE and CTRP data based on the cell line ID and calculate correlations

cor_CTRP <- left_join(CCLE_pan,

   CTRP_pan %>% select(ID, any_of(Drugs)), by = "ID") %>%

 na.omit() %>%

 select(-ID, -Lineage, -CCLE_Name) %>%

 cor() %>%

 as_tibble(rownames = "gene") %>%

 select(gene, any_of(Drugs)) %>%

 filter(!(gene %in% Drugs))

cor_analysis <- cor_CTRP %>%

 mutate(r_AUCs = (erastin + ML210 + ML162) / 3) %>%

 mutate(group = ifelse(gene %in% gene_list[["Top_ranking_genes"]], "Top Ranking", "All genes"))

 rowwise() %>%

 mutate(x = runif(n = 1)) %>%

 ungroup()

#Visualization of correlation analysis

ggplot(cor_analysis, aes(y = r_AUCs, x = x)) +

 geom_point(data = thisFig %>% filter(group == "All genes"), aes(color = group, alpha = group)) +

 geom_point(data = thisFig %>% filter(group != "All genes"), aes(color = group, alpha = group)) +

 scale_color_manual(values = c("gray", "red")) +

 scale_alpha_manual(values = c(0.2, 0.8)) +

 geom_violin(data = thisFig %>% filter(gene %in% gene_list[["Top_ranking_genes"]]), fill = "salmon", alpha = 0.2) +

 geom_hline(yintercept = 0, linetype = "dashed") +

 labs(x = "", y = "Correlation to mean FIN AUCs") +

 theme_classic(base_size = 18) +

 theme(legend.position = "none",

   axis.text = element_text(color = "black"),

   axis.ticks.x = element_blank(),

   axis.text.x = element_blank())

#Calculate enrichment scores and p-values using FGSEA

ranks_CTRP <- setNames(cor_analysis$r_AUCs, cor_analysis$gene)

FGSEA_CTRP <- fgsea(pathways = gene_list, stats = ranks_CTRP, maxSize = 1000, nproc = 0, scoreType = "std")

#Plot enrichment for the top-ranking genes

plotEnrichment(gene_list[["Top_ranking_genes"]], ranks_CTRP)

## Limitations

In this protocol we combine two computational drug discovery approaches, to increase the confidence and predictive power of each approach. Still, the pipeline is based on correlations and thus may result in some false positives. In our experience, the rate of false positives is extremely small. In our paper,^1^ 9 genes out of the 10 potential genes selected for experimental validation proved indeed to be ferroptosis repressors. It is, therefore, a good practice to select several potential targets. Further, while the ranking of the predictors by the “nearest neighbors” method had very good results, it relies on pre-existing knowledge concerning the pathway, which is not always available.

## Troubleshooting

### Problem 1

The transcriptomic similarity scores calculated in step 9 are too variant. This is usually a result of the variant response signature of the drugs in different cell lines, a well-known phenomenon (see Baillif et al.^6^). This may introduce some noise in the ranked lists obtained in steps 13 and 15.

### Potential solution

The pipeline may benefit by introducing a predictor selection step. To that end, the drugs response signatures in the different cell lines (obtained in step 5) are compared to each other, and those that are far away from the consensus of all others are flagged. The transcriptomic similarity scores associated with the flagged signatures are removed from the analysis done in the next steps. To calculate the distance of each response signature from the consensus, we use the fold changes values, determined in step 5. We perform pair-wise correlations between all signatures, and then for each signature we sum up its correlation scores with all other signatures (excluding negative values). This metric is then used to visualize the distance of each signature from the consensus. This method can be performed using the following code.

#Fold_changes - a table showing all genes (in rows) and treatments (in column),

#with fold change for each gene and each treatment (calculated by Limma)

cor_matrix_FC <- Fold_changes %>%

 select(-genes) %>%

 na.omit() %>%

 cor(method = "pearson")

#calculate weights

weights <- cor_matrix_FC %>%

 as_tibble(rownames = "signatures") %>%

 mutate(across(-signatures, ∼ifelse(.x == 1, 0, .x))) %>% #self-correlations are ignored

 mutate(across(-signatures, ∼ifelse(.x < 0, 0, .x))) %>% #negative correlations are ignored

 mutate(sum = rowSums(across(-signatures))) %>%

 mutate(weights = sum / sum(sum) ∗ 100) %>%

 select(signatures, weights) %>%

 arrange(weights) %>%

 mutate(signatures = fct_reorder(signatures, weights))

#Plot all weights. Signatures with low score should be removed from the analysis

ggplot(weights, aes(x = weights, y = signatures)) +

 geom_col(aes(fill = weights < 9)) +

 scale_fill_manual(values = c("green4", "gray")) +

 labs(x = "Degree of Similarity (%)", y = "") +

 theme_bw(base_size = 16) +

 theme(legend.position = "none",

 panel.grid = element_blank(),

 axis.text = element_text(color = "black", size = 14))

#plot the correlation matrix

matrix <- cor_matrix_FC %>%

 as_tibble(rownames = "signatures") %>%

 gather(-signatures, key = "treat", value = "R")

sorted_signatures <- levels(weights$signatures)

matrix$signatures <- factor(matrix$signatures , levels = sorted_signatures)

matrix$treat <- factor(matrix$treat, levels = sorted_signatures)

ggplot(matrix, aes(x = pert, y = treat, fill = R)) +

 geom_tile(color = "black") +

 geom_text(aes(label = round(R, 2)), size = 2) +

 scale_fill_gradient2(low = "blue", mid = "white", high = "green4") +

 scale_x_discrete(expand = c(0,0)) +

 scale_y_discrete(expand = c(0,0)) +

 labs(x = "", y = "") +

 theme_bw(base_size = 16) +

 theme(legend.position = "left",

   axis.text = element_text(color = "black"),

   axis.text.x = element_text(angle = 90, hjust = 1, vjust = 0.5))

### Problem 2

The UMAP approach (steps 14-15) does not yield good potential targets (the method described under “quantification and statistical analysis” does not yield significant enrichment).

### Potential solution

In our previous publication^1^ we showed that the UMAP approach for ranking genes is stable for the stochastic characteristics of UMAP, and that distances in the UMAP actually recapitulate distance in the predictor matrix. Still, if this approach fails based on the validation method described in the statistical analysis section, the following solutions may help:

• Change UMAP parameters: Specifically the min_dist parameter which affects distances between nodes on the UMAP.
• Use another dimension reduction methods: although the UMAP method has many advantages over other methods, we got good results (unpublished) by using PCA, followed by k-means clustering. Other dimension reduction methods can be used as well.
• The “best predictor” method (steps 11-13) can be used instead of the dimension reduction method.

## Resource availability

### Lead contact

Further information and requests for resources and reagents should be directed to and will be fulfilled by the lead contact, Sima Lev (sima.lev@weizmann.ac.il).

### Technical contact

Technical questions on executing this protocol should be directed to and will be answered by the technical contact, Yaron Vinik (yaron.vinik@weizmann.ac.il).

### Materials availability

This study did not generate new unique reagents.

### Data and code availability

The extended version of the code used in this protocol, including example data that can be used to reproduce this protocol, is given in Zenodo (https://doi.org/10.5281/zenodo.13370886) and GitHub (https://github.com/SimaLevLab/Pipeline-for-discovery-of-ferroptosis-targets).

## Acknowledgments

S.L. is the incumbent of the Joyce and Ben B. Eisenberg Chair of Molecular Biology and Cancer Research. This study was supported by Merck KGaA, Darmstadt, Germany.

## Author contributions

Y.V., conceptualization and writing; A.M., review; S.L., manuscript editing, supervision, and funding acquisition.

## Declaration of interests

The authors declare no competing interests.
